# Unexpected Occurrence of Plasmid-Mediated Quinolone Resistance Determinants in Environmental *Aeromonas* spp.

**DOI:** 10.3201/eid1402.070677

**Published:** 2008-02

**Authors:** Vincent Cattoir, Laurent Poirel, Camille Aubert, Claude-James Soussy, Patrice Nordmann

**Affiliations:** * Institut Nationale de la Santé et de la Recherche Médicale Unité 914, Le Kremlin-Bicêtre, France; † Hôpital de Bicêtre, Le Kremlin-Bicêtre; ‡ Université Paris XII, Créteil, France

**Keywords:** Qnr, QnrS, antibiotic resistance, reservoir, *Aeromonas punctata* subsp. *punctata*, *Aeromonas media*, mobile insertion cassette, research

## Abstract

We searched for plasmid-mediated quinolone resistance determinants of the Qnr type in several water samples collected at diverse locations from the Seine River (Paris, France). The *qnrS2* genes were identified from *Aeromonas punctata* subsp. *punctata* and *A. media*. The *qnrS2* gene was located on IncU-type plasmids in both isolates, which resulted in increased MIC values of quinolones and fluoroquinolones, once they were transferred into *Escherichia coli*. The *qnrS2* gene identified in *A. punctata* was part of novel genetic structure corresponding to a mobile insertion cassette element. This identification of plasmid-mediated *qnr* genes outside *Enterobacteriaceae* underlines a possible diffusion of those resistance determinants within gram-negative rods.

## Abstract 2

Identification of plasmid-mediated *qnr* genes outside Enterobacteriaceae underlines a possible diffusion of those resistance determinants within gram-negative rods.

Quinolones are broad-spectrum antibacterial agents used in human and veterinary medicine. Their extensive use has been associated with a rising level of quinolone resistance (*1*). The 2 main mechanisms of quinolone resistance are chromosomally encoded, either a modification of the quinolone targets with changes of DNA gyrase (*gyrA*) and/or of topoisomerase IV (*parC*) genes; or a decreased intracellular concentration due to impermeability of the membrane or to overexpression of efflux pump systems (*2*). Plasmid-mediated quinolone resistance was first identified in a *Klebsiella pneumoniae* clinical isolate from the United States (*3*). It is mediated by a 218-aa protein, Qnr (lately termed QnrA), which belongs to the pentapeptide repeat family of proteins that protects DNA from quinolone binding to topoisomerases (*4*,*5*). QnrA confers resistance to quinolones such as nalidixic acid and increases MICs of fluoroquinolones up to 32-fold in *Escherichia coli* (*6*). In addition, it enhances selection of associated chromosome-encoded quinolone resistance determinants that confer additional resistance to fluoroquinolones (*7*). The QnrA determinants have been reported worldwide in many enterobacterial species, and 6 of them are known so far (QnrA1 to QnrA6) (*8*). Other plasmid-mediated quinolone resistance determinants, QnrB (QnrB1 to QnrB10) and QnrS (QnrS1 and QnrS2), have been identified in enterobacterial species, sharing 41% and 60% amino acid identity with QnrA, respectively (*8*–*10*). The plasmid-mediated *qnr* genes have been identified so far only in *Enterobacteriaceae* (*6*,*8*). Recent findings indicated that those genes originate from environmental gram-negative bacterial species, such as *Shewanella algae,* the progenitor of the *qnrA* genes (*11*), and *Vibrio splendidus,* the progenitor of *qnrS* genes (*12*). We have shown that many *Vibrionaceae* species may harbor chromosome-encoded *qnr*-type genes (*13*).

To further evaluate the spread of plasmid-mediated resistance determinants in the environment, we have searched for those genes in water samples drawn from the Seine River in Paris, France. We identified QnrS determinants in *Aeromonas* species in uncommon genetic environments.

## Materials and Methods

### Water Sampling and Screening for *qnr* Genes

Water samples (40 mL each) were collected in November 2006, ≈0.2 m below the water surface, by immersion of 50-mL sterile, screw-capped tubes. Six distinct urban sites located on the Seine River in Paris were sampled: center (site 1), north (site 2), east (site 3), south (site 4), and west (sites 5 and 6). The Seine crosses the city of Paris (2. 4 million inhabitants) from southeast to northwest.

Samples were stored at 4°C before DNA extraction. To estimate the total number of viable bacteria, we diluted all samples in 0.85% saline solution and plated them (0.2 mL) on chromogenic URISelect4 agar (Bio-Rad, Marnes-la-Coquette, France) without any antimicrobial agent, which allowed visual differentiation of gram-negative species. After enrichment of 1 mL of water sample in 10 mL of trypticase soy (TS) broth for 48 h at 30°C and 37°C, *qnrA*-, *qnrB*- and *qnrS* genes were detected by using a multiplex PCR-based strategy (see below).

### Isolation and Identification of *qnr-*Positive Isolates

To identify the *qnr*-positive isolates, 1 mL of each *qnr*-positive water sample was grown in 10 mL of TS broth containing nalidixic acid (16 mg/L) for 24–48 h at 30°C and 37°C. After dilution in 0.85% NaCl, each broth was plated (0.2 mL) on chromogenic URISelect4 agar and 5% horse-blood TS agar, both containing nalidixic acid (16 mg/L). Finally, each colony was tested by multiplex PCR for detection of *qnr* genes (see below). Further identification of *qnr*-positive isolates was performed by conventional biochemical techniques (API-20E and API-NE systems [bioMérieux, Marcy-l’Etoile, France]), and by sequencing of 16S rDNA and *gyrB* genes, as previously described (*14*,*15*).

### Isolation of Total DNA and PCR Amplification

Whole-cell DNA was extracted from water samples by using QIAamp DNA Mini Kit (QIAGEN, Courtaboeuf, France), according to the manufacturer’s recommendations. Whole-cell DNA from isolated colonies identified after culture was extracted by using the boiling technique, which includes a heating step at 100°C of a single colony in a volume of 100 μL of distilled water followed by centrifugation of the cell suspension.

Screening of the *qnrA-*, *qnrB-*, and *qnrS* genes in water samples and from isolated colonies was conducted by using a multiplex PCR-based technique able to amplify the known Qnr variants, as previously described (*16*). *E. coli* Lo, *K. pneumoniae* B1, and *E. coli* S7 strains were used as *qnrA*-, *qnrB*-, and *qnrS-*positive controls, respectively (*16*,*17*). PCR amplicons were sequenced on both strands (see below). The chromosome-encoded quinolone-resistance determining regions (QRDRs) of *gyrA* and *parC* genes were sequenced after PCR amplification by using whole-cell DNA of *qnr*-positive isolates, as previously described (*18*).

PCR assays were also performed by using standard conditions of amplification (*17*) to detect genes coding for the replication protein of IncU-type plasmids (*rep* gene) and the tetracycline resistance determinant (encoded by the *tetA* gene). The primers, designed for this study, are as follows: for *rep*, Rep-IncU-F (5′-CTGGCTGAAATGCTGTTGCC-3′) and Rep-IncU-R (5′-GCTTCATAGGCTTCACGCTC-3′) to give a 1,199-bp product, and for *tetA*, TetA-1 (5′-GTGAAACCCAACAGACCCC-3′) and TetA-2 (5′-TCAGCGATCGGCTCGTTGC-3′) to give a 589-bp product.

### Antimicrobial Drug–Susceptibility Testing

The antimicrobial drug susceptibility of *Aeromonas* isolates was first determined by the disk diffusion technique on Mueller-Hinton (MH) agar plates according to Clinical and Laboratory Standards Institute guidelines (*19*). The disks were supplied by Bio-Rad Laboratories, and the following antimicrobial agents were tested: amoxicillin (25 μg), ticarcillin (75 μg), amoxicillin-clavulanate (20/10 μg), ticarcillin-clavulanate (75/10 μg), piperacillin (75 µg), piperacillin-tazobactam (75/10 μg), cephalothin (30 μg), cefuroxime (30 μg), cefoxitin (30 μg), cefotaxime (30 μg), ceftazidime (30 μg), aztrem (30 μg), latamoxef (30 μg), cefepime (30 μg), imipenem (10 μg), kanamycin (30 IU), tobramycin (10 μg), gentamicin (15 μg), netilmicin (30 μg), amikacin (30 μg), nalidixic acid (10 μg), norfloxacin (5 μg), ofloxacin (5 μg), ciprofloxacin (5 μg), chloramphenicol (30 μg), tetracycline 30 (IU), fosfomycin (50 μg), rifampicin (30 μg), sulfonamide (200 μg), trimethoprim (5 μg), and colistin (50 μg).

Thus, MICs of quinolones and fluoroquinolones were determined by using the E test technique, according to the manufacturer’s recommendations (AB Biodisk, Solna, Sweden). MIC breakpoints were retained for determining susceptibility and resistance ranges to nalidixic acid and ciprofloxacin were <8 and >32 mg/L and <1 and >4 mg/L, respectively, as recommended for *Enterobacteriaceae* (*19*).

### Transfer of Resistance and Plasmid Analysis

Transfer of the plasmid-mediated quinolone resistance markers from *qnr*-positive *Aeromonas* isolates to *E. coli* TOP10 was attempted by using electroporation and conjugation techniques (*20*). *E. coli* TOP10 and azide-resistant *E. coli* J53 strains were recipient strains for transformation and conjugation experiments, respectively (*20*). Conjugation experiments were performed at 22°C and 37°C, as previously described (*21*). Transformants and transconjugants were selected on MH agar plates containing nalidixic acid (3 mg/L) only or containing azide (100 mg/L) plus nalidixic acid (6 mg/L), respectively. Plasmid extraction was performed from each *qnr*-positive isolate and its corresponding transformants by using the Kieser technique (*22*). Plasmid sizes were determined by electrophoresis on an agarose gel and comparison with sizes of reference plasmids (164, 66, 38, and 7 kb) of *E. coli* NCTC 50192, as previously described (*20*). All transformants were confirmed to be *qnr* positive by mulitplex PCR (see above).

Cloning experiments were performed with *Eco*RI-restricted whole-cell DNA of *Aeromonas* isolates 37 and 42, followed by ligation of DNA fragments in the *Eco*RI-site of cloning vector pBK-CMV. Recombinant plasmids were then transformed by electroporation into *E. coli* TOP10 electrocompetent cells. *E. coli* TOP10 harboring recombinant plasmids were selected on MH agar plates containing kanamycin (30 mg/L) and nalidixic acid (3 mg/L). All clones were tested as carrying *qnr* gene by multiplex PCR (see above), and cloned fragments of recombinant plasmids were sequenced by primer walking (see below).

### Sequencing and Bioinformatic Analysis

PCR products, purified with a Qiaquick PCR Purification Kit (QIAGEN), and recombinant plasmids were sequenced by using an Applied Biosystems sequencer (ABI377, Foster City, CA, USA). The nucleotide sequences and the deduced protein sequences were analyzed with BLAST software (www.ncbi.nlm.nih.gov/BLAST).

## Results

### QnrS2 Determinant from *Aeromonas* spp.

Samples extracted from 2 of the 6 sites (sites 3 and 4) from the Seine River in autumn 2006 were PCR positive for *qnrS* genes. Sequencing of the amplicons identified a *qnrS2* gene in both cases. Further samples were therefore collected 1 week later from the same collecting sites and assessed the permanent occurrence of QnrS2- positive isolates in that river at that time. No *qnrA* and *qnrB* genes were detected.

The total number of gram-negative bacteria from water collection varied from 10^3^ to 10^5^ CFU per mL, depending on the sampling site, and included ≈10%–50% of isolates belonging to *Aeromonas* species (data not shown). The QnrS2-positive isolates were identified as *Aeromonas* species according to their phenotypic characterization. Strain 37 (site 4, pink colonies on URI4Select agar) and strain 42 (site 3, blue colonies on URI4Select agar) were selected as *qnrS2*-positive *Aeromonas* isolates to be further characterized. Genotypic identification was first attempted by sequencing of a ≈1,200-bp fragment of the 16S rDNA gene, but sequence analysis did not allow a precise identification. Isolate A37 was identified as *A. trota* or *A. punctata* (formerly *A. caviae*) (only 1 nt difference) and isolate A42 as *A. hydrophila* or *A. media* (only 1 nt change). Because *gyrB* sequence divergence is greater than that of 16S rDNA, phylogenetic analysis based on *gyrB* sequence (allowing more reliable identification of members of the genus *Aeromonas* [*15*]) identified isolate A37 as *A. punctata* subsp. *punctata* and isolate A39 as *A. media*.

The *Aeromonas* isolates both displayed a wild-type resistance phenotype to β-lactams with resistance to narrow-spectrum penicillins and remained susceptible to several β-lactam molecules, including broad-spectrum cephalosporins and carbapenems. *A. media* 42 was also resistant to several aminoglycosides (kanamycin, tobramycin), chloramphenicol, and tetracycline, whereas *A. punctata* 37 was fully susceptible.

*Aeromonas* isolates 37 and 42 were resistant to nalidixic acid (MIC >256 mg/L) and to fluoroquinolones (Table). However, *A. media* 42 exhibited higher resistance levels to fluoroquinolones, with MICs 2- to 8-fold higher than those for *A. punctata* 37 (Table). Sequence analysis of the QRDR regions of *gyrA* and *parC* genes showed that *A. punctata* 37 had 1 aa substitution, Ser83Ile in GyrA, whereas *A. media* 42 had 2 aa substitutions, Ser83Ile in GyrA and Ser80Ile in ParC, as compared with the wild-type proteins of *Aeromonas* species (*18*).

**Table:** MICs (mg/L) of quinolones and fluoroquinolones for *Aeromonas punctata* 37, *A. media* 42, and *Escherichia coli* TOP10 harboring natural plasmids p37 and p42*

Antimicrobial agent	*A. punctata 37*	*A. media* 42	*E. coli* TOP10 with plasmids		*E. coli* TOP10
			p37	p42	
Nalidixic acid	>256	>256	4	4	1
Norfloxacin	16	64	1	1	0.03
Ofloxacin	8	>32	0.5	0.5	0.01
Ciprofloxacin	4	>32	0.12	0.25	<0.01
Moxifloxacin	4	32	0.25	0.25	<0.01
Sparfloxacin	16	>32	0.25	0.25	<0.01
Enrofloxacin	4	>32	0.5	0.5	<0.01

*Each expressing the plasmid-mediated quinolone determinant QnrS2 from *A. punctata* 37 and *A. media* 42, respectively, and *E. coli* reference strain TOP10.

### *qnrS2* Gene on IncU-Plasmid Backbone

**Figure 1:**
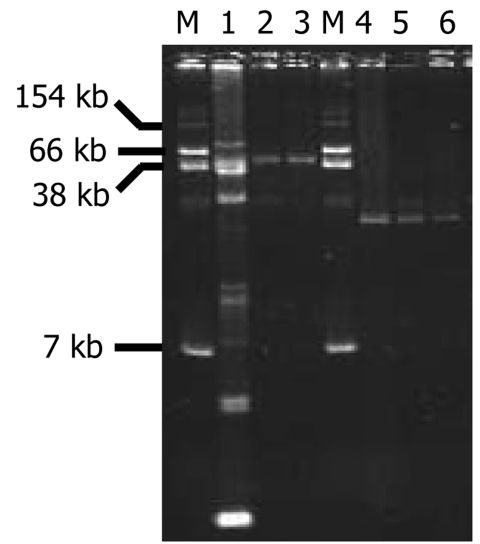
Plasmid DNAs from *Aeromonas punctata* 37 and *A. media* 42 and their *Escherichia coli* TOP10 transformants (TF) carrying plasmids p37 or p42. Lanes: 1, *A. punctata* 37; 2, *E. coli* TOP10/p37 TF-1; 3, *E. coli* TOP10/p37 TF-2; 4, *A. media* 42; 5, *E. coli* TOP10/p42 TF-1; 6, *E. coli* TOP10/p42 TF-2; M, *E. coli* NCTC 50192 (used as reference for plasmid sizes).

The plasmid-mediated quinolone resistance QnrS2 determinant was transferred from *Aeromonas* isolates 37 and 42 to *E. coli* TOP10 recipient strain by electrotransformation, but repeated conjugation experiments failed. Plasmid analysis identified a single 55-kb plasmid (p37) and a single 20-kb plasmid (p42) from *E. coli* transformants from *A. punctata* 37 and *A. media* 42 isolates, respectively (Figure 1). After they were transferred into *E. coli* TOP10, plasmids p37 and p42 conferred increased MICs of quinolones and fluoroquinolones (Table). No other antimicrobial resistance marker was carried by those natural plasmids.

**Figure 2:**
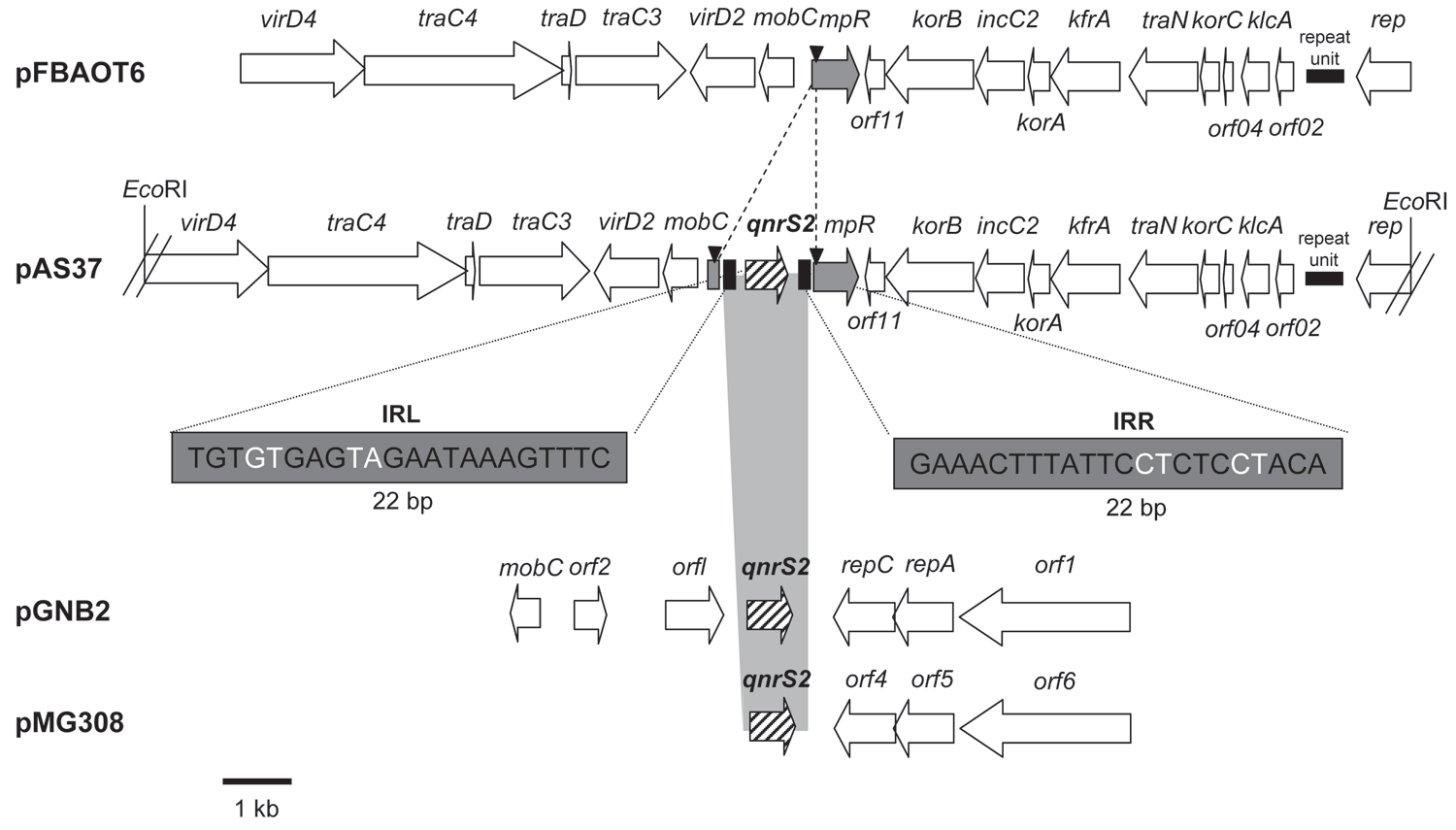
Genetic environments of the *qnrS2* gene in plasmid p37 from *Aeromonas punctata* 37 and comparison with related plasmid structures. Plasmid pFBAOT6 is from *A. punctata* from the United Kingdom (*23*); plasmids pGNB2 and pMG308 are from a wastewater treatment plant from Germany (unknown bacterial reservoir) (*24*) and from a non-Typhi *Salmonella* clinical isolate from the United States (*25*), respectively. Recombinant plasmid pAS37 has been obtained from our study. Open reading frames (ORFs) are indicated by horizontal arrows. The right and left inverted repeats (IRR and IRL) are indicated, and duplication sites (CCTCC) are represented by black triangles. The *Eco*RI- restriction sites that have been used for cloning experiments are indicated. The identified mobile insertion cassette element is bracketed by IRL and IRR of 22-bp size (bases in black are identical, and bases in white are different).

Cloning of *Eco*RI-restricted DNA from whole-cell DNA of *A. punctata* 37 produced a recombinant plasmid, pAS37, containing a 19,050-bp insert that contained the *qnrS2* gene. Sequencing showed that the *qnrS2* gene was located in a plasmid-encoded genetic structure previously identified in an IncU-related plasmid, pFBAOT6 (84,748 bp), isolated from an *A. punctata* strain recovered from hospital sewage in Kendal (United Kingdom) in 1997 (*23*). This region consisted of 15 open reading frames (Figure 2). Detailed analysis of pAS37 showed that a fragment of 1,375 bp containing the *qnrS2* gene (657 bp) was inserted within the *mpR* gene coding for a putative zinc metalloprotease (MpR) (Figure 2). Sequencing of the full insert in plasmid pAS37 identified the same *rep* region as reported from the IncU-related pFBAOT6 plasmid, which indicated that p37 was a member of the IncU incompatibility group. PCR amplification that used specific primers of this *rep* gene also gave positive results from whole-cell DNA of *E. coli* (p42) transformant, indicating that p42 also belonged to IncU-type plasmid family. In addition, cloning of *Eco*RI-restricted DNA from whole-cell DNA of *A. media* 42 showed that the *qnrS2* gene was inserted into the *mpR* gene in plasmid p42. No *tetA* gene encoding resistance to tetracycline was detected by PCR in plasmids p37 and p42, whereas it was identified in plasmid pFBAOT6 (*23*).

### *qnrS2* Gene as Part of a Mobile Insertion Cassette

The *qnrS2* gene was identified inside a 1,375-bp structure bracketed by two 22-bp imperfect inverted repeats (4 mismatches) (Figure 2). This structure ressembled a transposon; however, no transposase encoding gene was associated with the *qnrS2* gene, the single gene identified in this genetic structure. Acquisition of that structure was likely the result of a transposition process because it was bracketed by a 5-bp duplication of the target site (CCTCC) that might be likely considered as the signature of transposition. Thus, this genetic structure defines a mobile insertion cassette (mic) containing the *qnrS2* gene instead of a transposase, as observed in insertion sequences. Those mic elements are not related to the gene cassette associated with class 1 integrons (*26*). The mic-*qnrS2* element identified here carried putative promoter sequences able to enhance the *qnrS2* expression made of a –35 box (TTCTCT) and a –10 box (TAACTT) separated by a 17-bp sequence.

## Discussion

Our study identified plasmid-mediated quinolone resistance QnrS determinants from water samples collected in different sites in a Paris river. To our knowledge, this is the first identification of plasmid-mediated QnrS determinants in nonenterobacterial species. Previous studies did not identify such *qnr* genes from tested gram-negative isolates that represented *Campylobacter jejuni* (*27*), *Aeromonadaceae*, *Pseudomonadaceae*, *Xanthomonadaceae*, *Moraxellaceae*, and *Shewanellaceae* (*11*). Identification of QnrS-positive isolates at 2 collection sites in different water samples may highlight their relative persistence in the environment, at least in this area at that time.

The high-level resistance to quinolones and fluoroquinolones might be due to mutations in type II topoisomerase genes because the mutations described in type II topoisomerases in *A. media* and *A. punctata* subsp. *punctata* have already been associated with resistance in *Aeromonas* spp (*18*). QnrS2 may confer low-level resistance to quinolones, as known in *E. coli*.

The *qnrS1* gene has been identified now from several enterobacterial isolates from Japan (*9*), Germany (*28*), the United Kingdom (*29*), the United States (*25*), France (*17*), Vietnam (*30*), Taiwan (*31*,*32*), and Denmark (*33*). The *qnrS2* gene (92% amino acid identity with QnrS1) was identified from a transferable IncQ-related plasmid (pGNB2) isolated from an activated sludge bacterial community of a wastewater treatment plant in Germany (*24*) and in a single non-Typhi *Salmonella* clinical isolate from the United States (*25*). Identification of QnrS determinants in *Aeromonas* spp. indicates that those bacterial species may play a role as a reservoir of the *qnrS* genes in an aquatic environment, as already evidenced for *tet* genes (*34*,*35*). However, whether *Aeromonas* species are a main or an accessory reservoir of plasmid-mediated quinolone resistance determinants in regard to *Enterobacteriaceae* remains to be determined. For *Aeromonas* spp. to act as a reservoir of *qnr* genes it must be capable of acquiring these resistance genes from their progenitors (*36*) and transferring this genetic information to *Enterobacteriaceae*. QnrS2-positive plasmids p37 and p42 were not able to be transferred by conjugation to an *E. coli* host in vitro, but they were able to replicate in *E. coli*, indicating their broad host spectrum. In addition, our study demonstrated that this IncU-type plasmid-mediated *qnrS2* gene was expressed and able to confer reduced susceptibility to quinolones, at least in *E. coli*. *Aeromonas* species and IncU plasmids, which are ubiquitous in a wide range of environments, might therefore act as important vectors for transfer of plasmid-mediated quinolone resistance determinants (*23*).

As opposed to most *qnrA* and *qnrB* genes, *qnrS* genes have never been reported to be associated with *sul1*-type class 1 integrons (*6*,*8*). The *qnrS1* gene has been identified either upstream of Tn*3*-like transposon (*9*,*28*) or upstream of the insertion sequence IS*Ecl*2 (*17*,*37*). In IncQ-related plasmid pGNB2 and in pMG308 from a *Salmonella* isolate, surrounding genetic structures of the *qnrS2* genes were similar, with 2 open reading frames located immediately downstream of *qnrS2,* similar to *repC* and *repA* genes involved in plasmid replication (*24*,*25*) (Figure 2). In plasmid p37 from *A. punctata* 37, the genetic structure was different since the *qnrS2* was part of a transposon-like structure and inserted in an open reading frame coding for a zinc MpR.

We have shown that plasmid integration of the *qnrS2* gene may result from a peculiar transposition process that likely corresponds to *trans*-transposition. The *qnrS2* gene was inserted in a peculiar mic. Such mic elements have been identified rarely, e.g., mic*231-*like elements in *Bacillus cereus*, carrying in only 1 instance an antibiotic resistance gene, the *fos* gene encoding resistance to fosfomycin (*26*). This finding may indicate that mic elements might be clinically relevant and the origin of an additional gene plasticity in a bacterial species. These elements containing genetic features involved in gene dissemination (with a transposase likely acting *in*-*trans*) and expression may be also vehicles for antibiotic resistance genes. This structure type may be added to the list of genetic tools at the origin of dissemination and expression of antibiotic resistance determinants.

As previously described for the spread of a carbapenemase gene (*bla*_IMI-2_) in US rivers (*20*), this report underlines that the aquatic environment is an important reservoir of novel antibiotic resistance determinants. Quinolones are antimicrobial agents extensively used in aquaculture and are stable molecules in water (as opposed to β-lactams) (*38*). Thus, they may be the source of an important driving force for selection of quinolone resistance, which explains why QnrS2-positive plasmids did not possess any additional resistance determinants. Further studies might focus on the particular effect of quinolone use for inducing the *qnrS2* gene mobility because it is known those molecules may induce bacterial repair systems and antibiotic resistance gene transfer (*39*).

We have previously shown that the *qnrA* and *qnrS* genes originate from water-borne bacterial species, *S. algae* and *Vibrio splendidus*, respectively. This identification of a *qnrS* gene in another water-borne species, *Aeromonas,* further strenghens the role of water as a vehicle for spread of those resistance determinants.
